# Supplementary material for: The interactome of intact mitochondria by cross-linking mass spectrometry provides evidence for coexisting respiratory supercomplexes
Source: Mol Cell Proteomics. 2017 Dec 8;17(2):216–32. doi: 10.1074/mcp.RA117.000470 (PMC5795388; doi:10.1074/mcp.RA117.000470)
Supplement: Supplemental Data [file supp_RA117.000470_133922_0_supp_23978_fzffwf.zip › spectra_annotation/mito_DR_spectra_annotation/308-1-9-1-11-1.pdf]

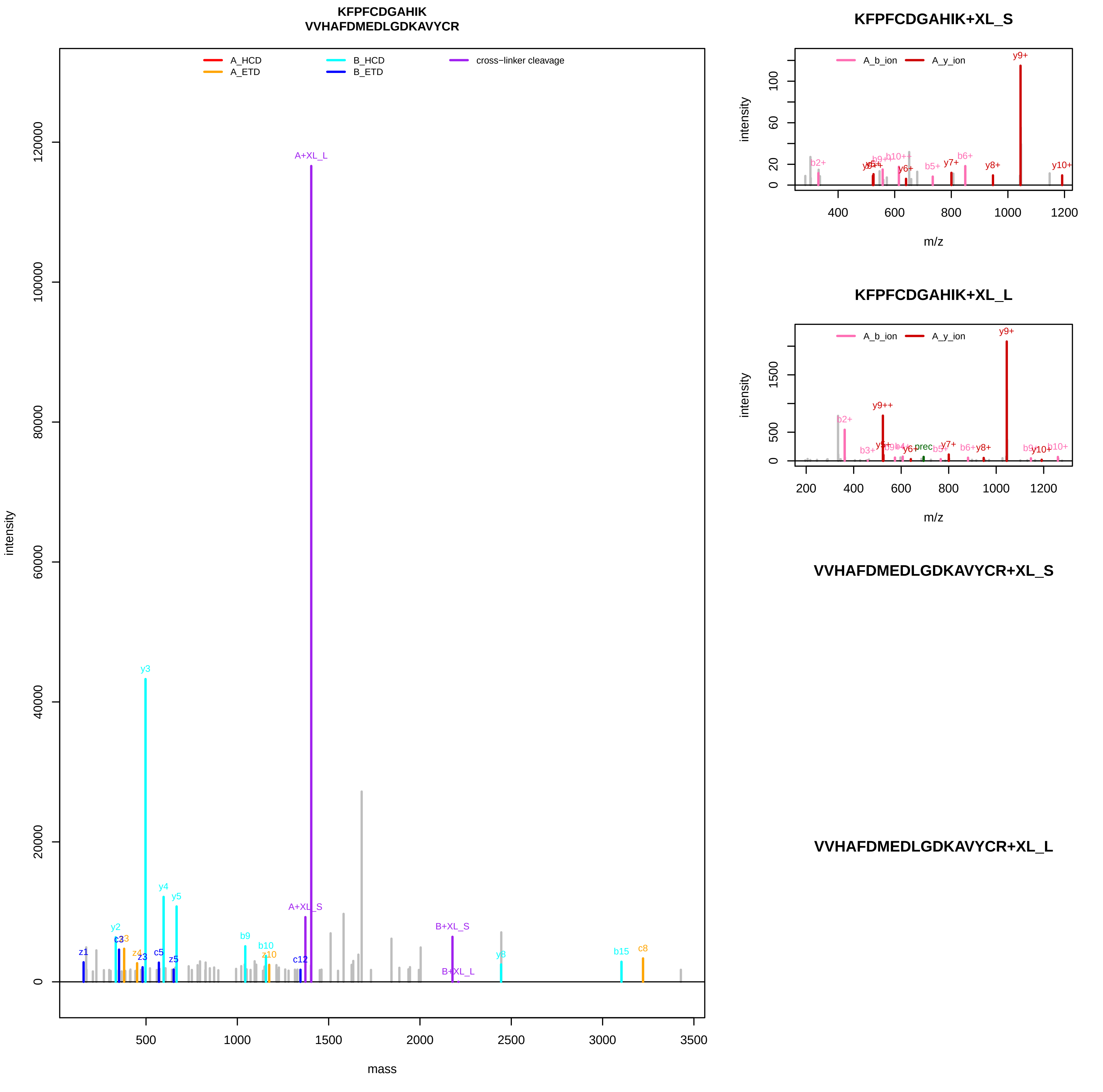
KFPFCDGAHIK
VVHAFDMEDLGDKAVYCR
A_HCD
A_ETD
B_HCD
B_ETD
cross−linker cleavage
A+XL_L
A+XL_S
B+XL_S
B+XL_L
y2
y3
y4
y5
y8
b9
b10
b15
c3
c5
c8
c12
z1
z3
z4
z5
z10
intensity
mass
0
20000
40000
60000
80000
100000
120000
500
1000
1500
2000
2500
3000
3500
KFPFCDGAHIK+XL_S
A_b_ion
A_y_ion
b2+
b5+
b6+
y6+
y7+
y8+
y9+
y10+
intensity
m/z
KFPFCDGAHIK+XL_L
A_b_ion
A_y_ion
y9+
y9++
b2+
b3+
y5+
y6+
prec
b5+
y7+
b6+
y8+
y10+
b10+
intensity
m/z
VVHAFDMEDLGDKAVYCR+XL_S
VVHAFDMEDLGDKAVYCR+XL_L
